# Supplementary material for: Microbial-Based Biofungicides Mitigate the Damage Caused by Fusarium oxysporum f. sp. cubense Race 1 and Improve the Physiological Performance in Banana
Source: J Fungi (Basel). 2024 Jun 12;10(6):419. doi: 10.3390/jof10060419 (PMC11204473; doi:10.3390/jof10060419)
Supplement: Supplementary file 1 [file jof-10-00419-s001.zip › jof-3022841-supplementary.pdf]

Supplementary material

Microbial-based biofungicides mitigate the damage caused by *Fusarium oxysporum* f. sp. *cubense* race 1 and improve the physiological performance in banana

Luisa Fernanda Izquierdo-García *, Sandra Lorena Carmona-Gutiérrez, Carlos Andrés Moreno-Velandia *, Andrea del Pilar Villarreal-Navarrete, Diana Marcela Burbano-David, Ruth Yesenia Quiroga-Mateus, Magda Rocío Gómez-Marroquín, Gustavo Adolfo Rodríguez-Yzquierdo and Mónica Betancourt-Vásquez

Corporación Colombiana de Investigación Agropecuaria, AGROSAVIA, Centro de Investigación Tibaitatá, Km 14 vía Bogotá a Mosquera, Mosquera, Cundinamarca, Colombia.

* Correspondence: lfizquierdo@agrosavia.co; cmoreno@agrosavia.co; Tel.: +57 601 4227373 EXT 1303

Abstract: Fusarium wilt of banana (FWB) is the most limiting disease in this crop. The phytosanitary emergency caused by FWB since 2019 in Colombia has required the development of ecofriendly control methods. The aim of this study was to test the effectiveness of microbial-based biofungicides against FWB caused by *Fusarium oxysporum* f. sp. *cubense* race 1 (Foc R1) and correlate such effect with plant physiological parameters. Five *Trichoderma* spp. (T1 to T4 and T9) and four *Bacillus* spp. (T5 to T8)-based biofungicides were evaluated in pot experiments. *In vitro* dual confrontation tests also were carried out to test whether the *in vitro* effects on Foc growth were consistent with *in vivo* effects. While *Trichoderma*-based T3, T4, and T9, and *Bacillus*-based T8 significantly reduced the growth of Foc R1 *in vitro, Trichoderma*-based T1, T3, T4 and T9 reduced temporarily Foc population into the soil. However, the incidence progress of FWB was significantly reduced by Bacterial-based T7 (74 % efficacy) and *Trichoderma*-based T2 (50 % efficacy). The molecular analysis showed that T7 prevented the inner tissue colonization by Foc R1 in 80 % of inoculated plants. T2, T4, T7, and T9 treatments mitigated the negative effects caused by Foc R1 on plant physiology and growth. Our data allowed to identify three treatments promising to control FWB reducing progress of the disease, delaying the colonization of inner tissue, and mitigating the physiological damages. Further studies should be addressed to determine the modes of action of the biocontrol agents against Foc and validate the utilization in the field.

Keywords: Biological control; Microbial consortia; Bioproducts; Panamá disease.

Table S1. Severity scale of the Fusarium wilt of banana*

Level of severity	Description of symptoms
0	Healthy plant, without symptoms
1	Yellowing, starting loss of turgor mostly in lower leav
2	Yellowing, loss of turgor or necrosis in all lower leaves with slight discoloration in young leaves
3	All leaves with intense yellowing, loss of turgor, epinasty
4	Death plant

* Modified from Dita et al. (2011). Dita, M.A., Waalwijk, C., Paiva, L.V., Souza, Jr M.T., Kema, G.H.J. (2011). A greenhouse bioassay for the *Fusarium oxysporum* f. sp. *cubense* x 'Grand Naine' (Musa, AAA, Cavendish subgroup) interaction. Acta Hort. 897, 377-380. https://doi.org/10.17660/ActaHortic.2011.897.51

Figure S1. Colony Forming Units (CFU) of the active ingredient per gram or per milliliter of each bioproduct according to the quality control compared to the information in the label.

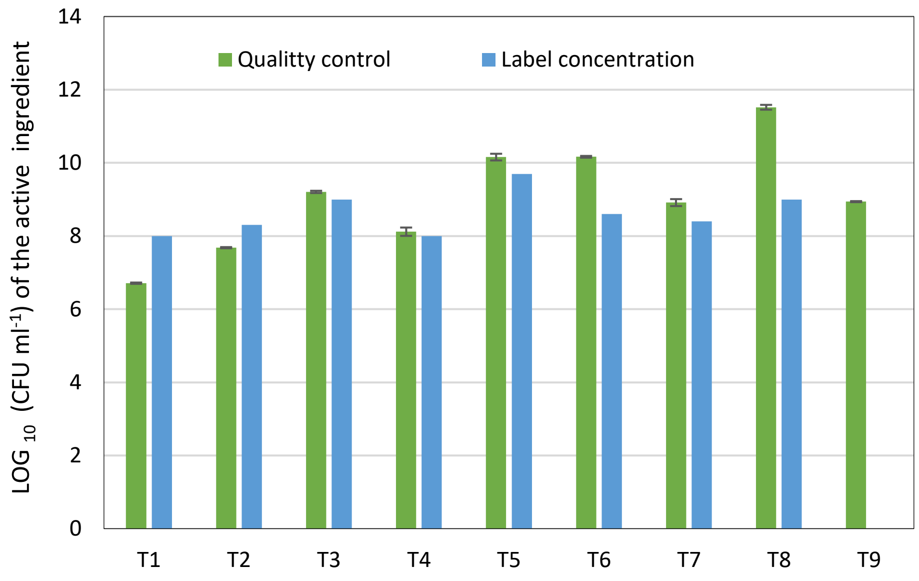

Figure S2. Progress curves of the disease intensity index (DII) for 72 to 77 days in each replicate of the entire experiment

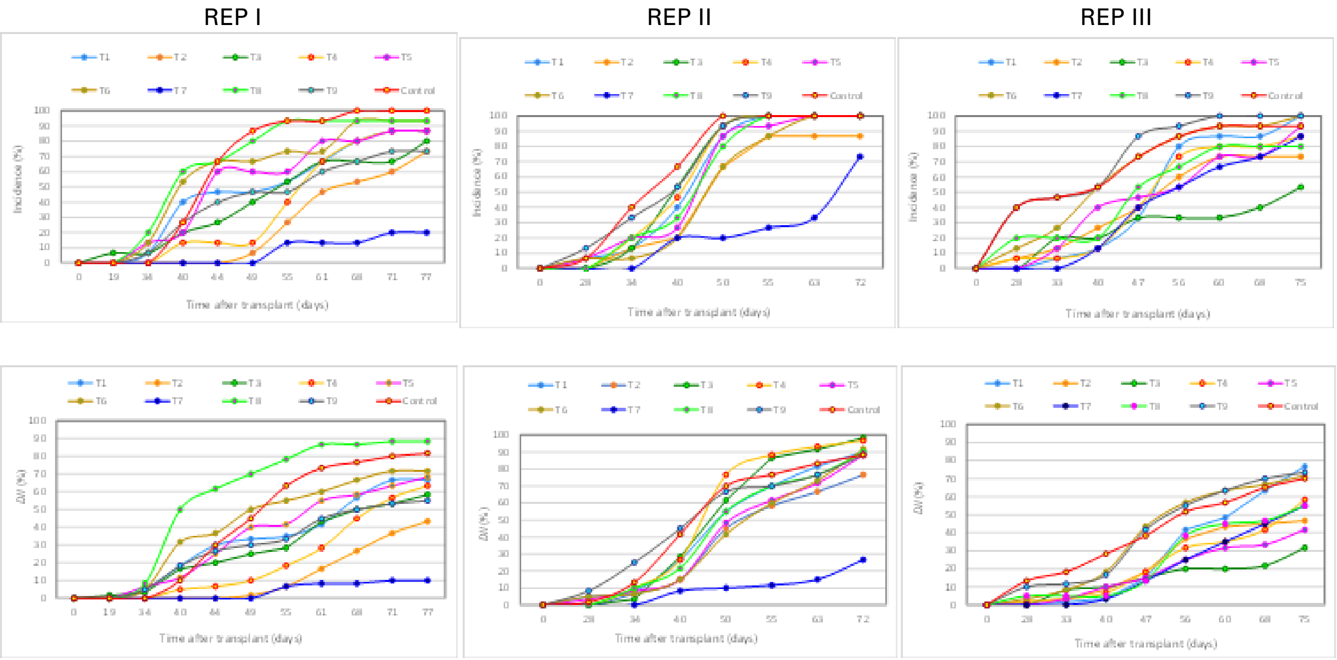

T1 – T9: Biofungicides. Control: negative control. REP: replicates of the experiment

Figure S3. Verification by electrophoresis in agarose gel (1.4%) of PCR amplification of the internal control BrepI (specific for repetitive elements of musaceous) on plant DNA from banana plants (replicate 2).

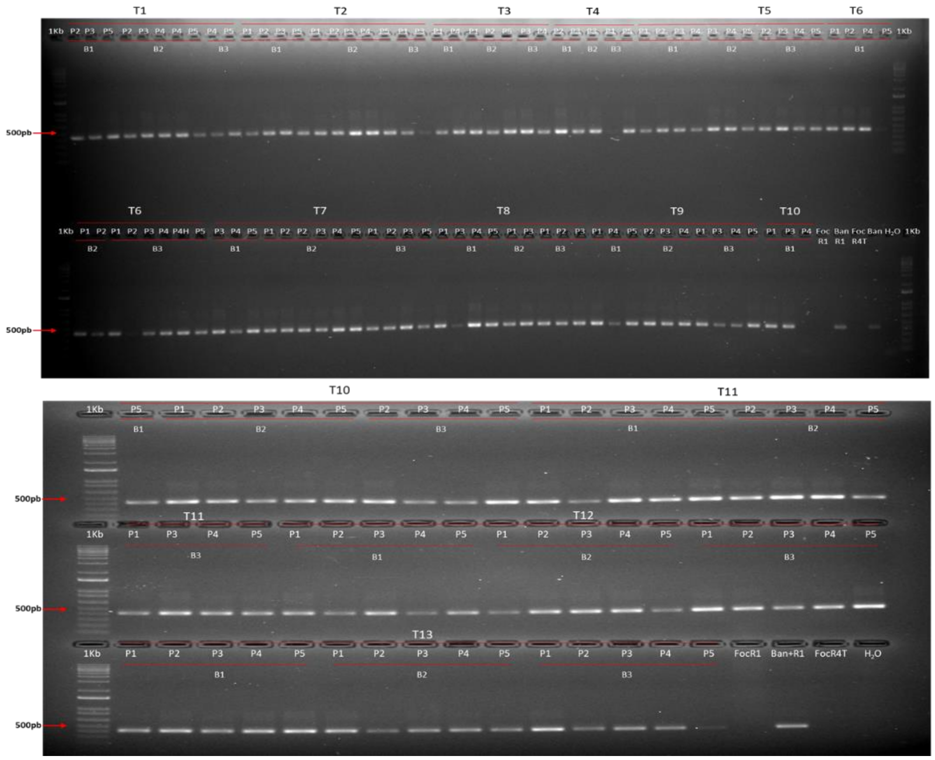

Figure S4. Verification by electrophoresis in agarose gel (1.4%) of PCR amplification of the marker SIX6b (specific for *Foc* R1) on plant DNA from banana plants (replicate 3).

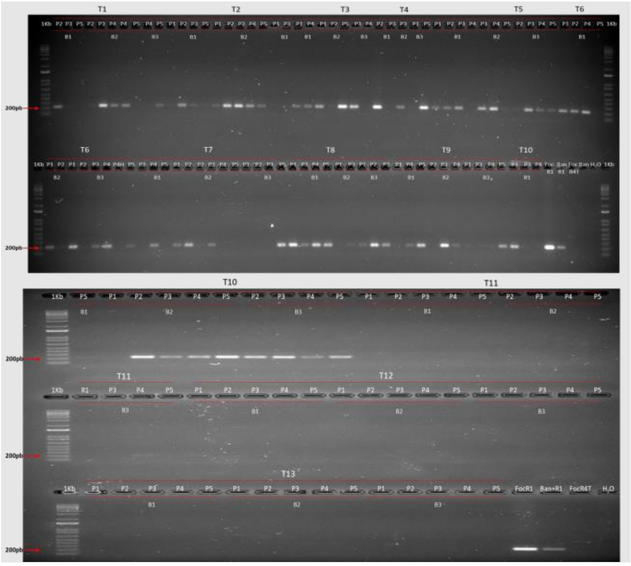

Table S2. Detection of *Foc* R1 in plant material from the replicate 1 of the *in vivo* experiment

**Treatment**	**Healthy plants (%)**	**Healthy plants negative for *Foc* R1 detection (%)**	**Healthy plants positive *Foc* R1 detection (%)**
T1	6.7	6.7	0.0
T2	13.3	13.3	0.0
T3	6.7	6.7	0.0
T4	0.0	0.0	0.0
T5	13.3	13.3	0.0
T6	6.7	0.0	6.7
T7	80.0	80.0	0.0
T8	0.0	0.0	0.0
T9	26.7	13.3	13.3
T10 (*Foc* R1)	0.0	-	-

Figure S5. Electron transport rate (ETR) recorded in banana plants at 49 (left panel) and 70 days after transplant (right panel) in soil artificially inoculated with *Foc* R1 and treated with biofungicides. Treatments sharing the same letter are not significantly different according to Fisher's minimum significant difference (LSD, α= 0.05). Each mean value is the result from three sampled plants (n=3).

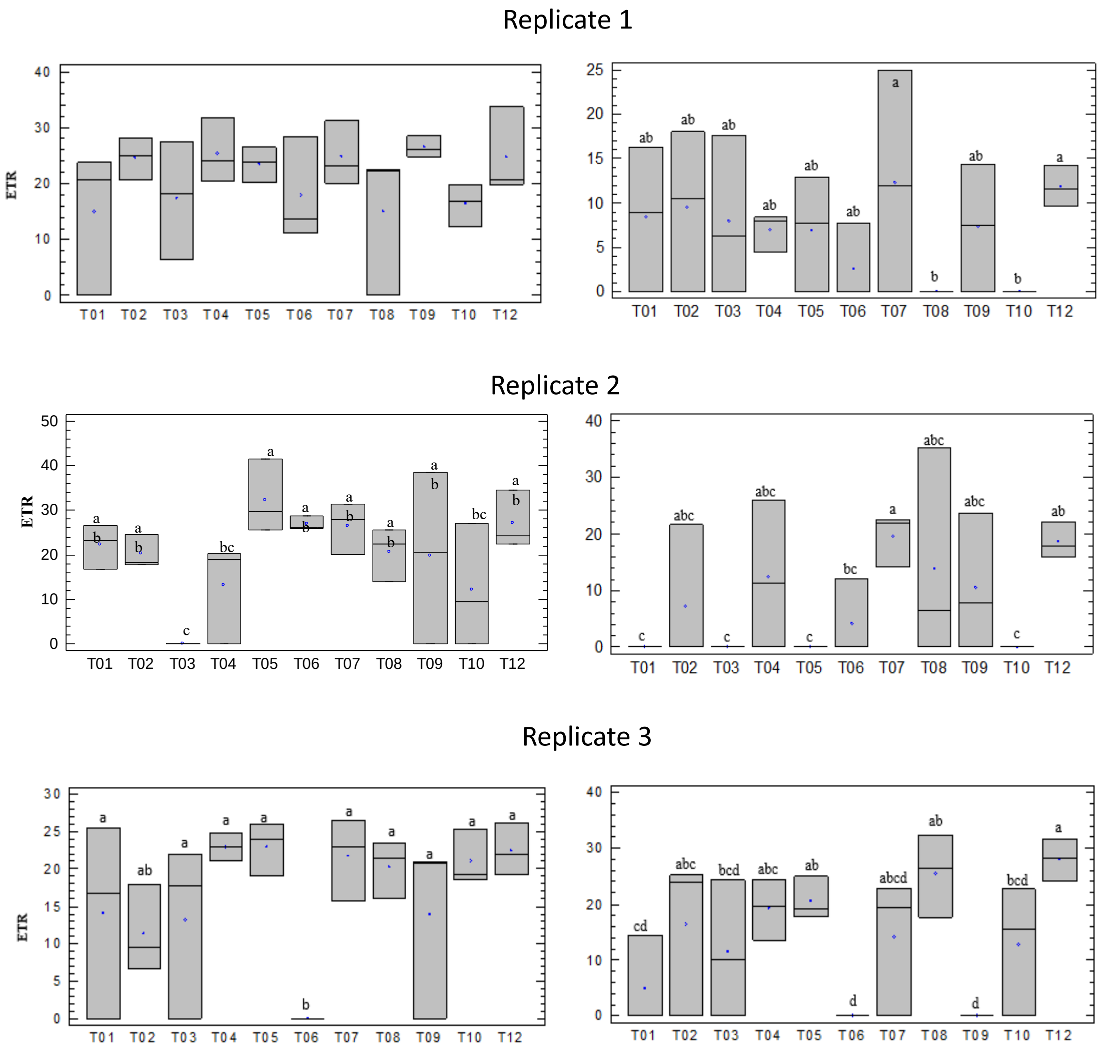

Table S3. Spearman´s correlation between disease severity index (SI) and physiological and growth variables

Physiological variables						
	**SI**	**A**	**E**	**ETR**	**Fv_Fm**	**Y_II**
**Replicate 1**						
**SI**		-0.6631	-0.3185	-0.8447	-0.8177	-0.8447
**A**	-0.6631		0.8085	0.7744	0.7448	0.7744
**E**	-0.3185	0.8085		0.3932	0.3622	0.3932
**ETR**	**-0.8447**	0.7744	0.3932		0.9125	1.0000
**Fv_Fm**	**-0.8177**	0.7448	0.3622	0.9125		0.9125
**Y_II**	**-0.8447**	0.7744	0.3932	1.0000	0.9125	
**Replicate 2**						
**SI**		-0.7164	-0.6735	-0.7099	-0.7632	-0.7099
**A**	-0.7164		0.9213	0.9249	0.9426	0.9249
**E**	-0.6735	0.9213		0.9091	0.885	0.9091
**ETR**	-0.7099	0.9249	0.9091		0.9374	1.0000
**Fv_Fm**	**-0.7632**	0.9426	0.885	0.9374		0.9374
**Y_II**	-0.7099	0.9249	0.9091	1.0000	0.9374	
**Replicate 3**						
**SI**		-0.4117	-0.4318	-0.6743	-0.7404	-0.6735
**A**	-0.4117		0.3806	0.6475	0.6211	0.6465
**E**	-0.4318	0.3806		0.6138	0.5726	0.6158
**ETR**	-0.6743	0.6475	0.6138		0.9093	0.9998
**Fv_Fm**	-0.7404	0.6211	0.5726	0.9093		0.9083
**Y_II**	-0.6735	0.6465	0.6158	0.9998	0.9083	

Growth parameters					
	**SI**	**Height**	**Diameter**	**REF**	**SPAD**
**Replicate 1**					
**SI**		-0.6726	-0.6929	-0.4247	-0.8134
**Height**	-0.6726		0.9301	0.5866	0.7897
**Diameter**	-0.6929	0.9301		0.6168	0.7857
**REF**	-0.4247	0.5866	0.6168		0.544
**SPAD**	**-0.8134**	0.7897	0.7857	0.5440	
**Replicate 2**					
**SI**		-0.4936	-0.6609	-0.5091	-0.8717
**Height**	-0.4936		0.9396	0.5252	0.7392
**Diameter**	-0.6609	0.9396		0.5839	0.8223
**REF**	-0.5091	0.5252	0.5839		0.6094
**SPAD**	**-0.8717**	0.7392	0.8223	0.6094	
**Replicate 3**					
**SI**		-0.7835	-0.7655	-0.3190	-0.8846
**Height**	-0.7835		0.8706	0.1902	0.7516
**Diameter**	-0.7655	0.8706		0.2050	0.7285
**REF**	-0.319	0.1902	0.205		0.32
**SPAD**	**-0.8846**	0.7516	0.7285	0.3200	

Figure S6. Growth of *Foc* race 1 colony on PDA medium in presence of biopesticide inoculum.

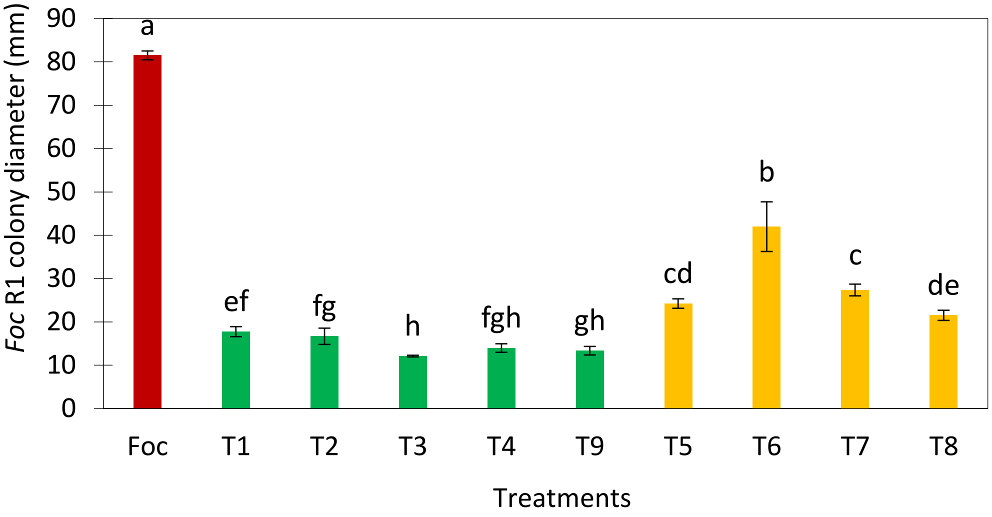

Bars on the columns indicate the standard deviation of the mean (n=3). Treatments sharing the same letter are not significantly different according to the Tukey's test (α= 0.05).

Table S4. Inhibition of diametral growth of the colony of *Foc* R1

Treatment	Inhibition (%)
T1	78.2 ± 1.14
T2	79,5 ± 2.13
T3	85,1 ± 0.11
T4	82,3 ± 0.21
T9	83,6 ± 1.28
T5	70,2 ± 1.29
T6	48,5 ± 6.85
T7	66,4 ± 1.63
T8	73,6 ± 1.27

± indicates standard deviation (SD) (n=3)
